# The (Mis)appropriation of HIV/AIDS advocacy strategies in Global Mental Health: towards a more nuanced approach

**DOI:** 10.1186/s12992-017-0263-3

**Published:** 2017-07-01

**Authors:** Alison Howell, China Mills, Simon Rushton

**Affiliations:** 1 Department of Political Science, Rutgers University, Newark, USA; 2 School of Education, University of Sheffield, Sheffield, UK; 3 Department of Politics, University of Sheffield, Sheffield, UK

**Keywords:** Global mental health, HIV/AIDS, International development, Global public health policy, Human rights, Medicalization

## Abstract

**Background:**

Mental health is increasingly finding a place on global health and international development agendas. Advocates for Global Mental Health (GMH), and international organizations such as the World Health Organization (WHO) and the World Bank, argue that treatments available in high-income countries should also be made available in low- and middle-income countries. Such arguments are often made by comparing mental health to infectious diseases, including the relative disease and economic burdens they impose, and pointing to the applicability of the right to access treatment for mental health, not only infectious diseases. HIV/AIDS advocacy in particular has been held up by GMH advocates as offering an appropriate strategy for generating global commitment.

**Discussion:**

There is a need to assess how health issues are framed not only in relation to social goods outside of health (such as human rights, security or development), but also in relation to other health or disease models, and how health policy and practice is shaped as a result. The article debates the merits and consequences of likening mental health to HIV/AIDS, and identifies four major problems with the model for GMH advocacy being developed through these analogies: 1. An inappropriately universalizing global approach to context-specific problems; 2. A conception of human rights that focuses on the right to access treatment at the expense of the right to refuse it; 3. A tendency to treat poverty as a psychiatric issue, rather than recognizing that mental distress can be the result of poverty and other forms of inequality; 4. The prioritization of destigmatization of disease over social justice models.

**Conclusion:**

There are significant problems with the wholesale adoption of an (often simplified) version of HIV/AIDS advocacy as a model for GMH. Yet critical engagement with the important and nuanced differences between HIV/AIDS and mental health may nevertheless point to some possibilities for productive engagement and cross-fertilisation between advocates, activists and scholars in both fields.

## Background

In recent years, the ‘Movement for Global Mental Health’ (MGMH) and international organizations such as the World Health Organization and the World Bank have put forward a case that ‘mental health is not only relevant to global health…but in fact lies at its very heart’ [1]. Global Mental Health (GMH) advocates have seen considerable success. For example, mental health is now included in the UN Sustainable Development Goals (SDGs) under Target 3.4 [2]. Key to this success has been the claim that mental health can and should be framed as a global health challenge similar to infectious disease, particularly HIV/AIDS. This comparison has been pursued in at least two ways.

Firstly, GMH advocates have compared both the disease burden and the economic burden of mental health to those of infectious disease, arguing that in comparison to infectious disease mental health is an even more pressing global problem. Secondly, GMH advocates have sought to learn from the success of HIV/AIDS advocacy, arguing that they should utilize similar strategies to gain attention and resources. They have argued that mental health should be framed as a truly ‘global’ problem, that an ethical case for the right to access to treatment in low and middle-income countries (LMICs) should be made, and that mental health should be a central goal within global health and development.

In this article, we raise the need for greater debate over the merits of this framing. We argue that GMH advocates are modelling advocacy on a selective and oversimplified version of the history of HIV/AIDS advocacy, and ignoring significant problems that result from falsely treating mental health and infectious disease as if they represent similar kinds of problem. We also suggest, however, that a more nuanced reading of the history of HIV/AIDS advocacy, which pays attention to its struggles and not only its successes, could provide some useful lessons for GMH advocates. In order to set the background for this debate, we begin with an account of the modeling of GMH advocacy on HIV/AIDS advocacy.

### Modeling GMH on HIV/AIDS advocacy: a brief history

HIV/AIDS has been the most prominent global health issue over the last 25 years and has captured the largest slice of development assistance for health. Effective advocacy has been crucial in generating this success [3–5]. AIDS activists from the early 1980s onwards famously fought stigma and discrimination, argued for a human right to access treatment, and called on governments in both the Global North and the Global South to increase their efforts to tackle the pandemic.

GMH advocates want to replicate this history in their own field. They have urged governments across the world - especially in LMICs- to take mental health more seriously, to agree there is ‘no health without mental health’ [6], and to scale up access to mental health services [7]. At the heart of current calls to ‘Mainstream mental health interventions into health, poverty reduction, development policies, strategies and interventions’ [8, 9] is the argument that, like HIV/AIDS, poor mental health is not only detrimental to individual wellbeing, but also to international development [9].

GMH advocates frequently make explicit comparisons between mental health and infectious diseases, especially HIV/AIDS. They argue that mental health occupies a low priority for policy makers and donors, compared to communicable diseases linked to premature mortality, and particularly to HIV/AIDS [10] despite estimates that ‘mental disorders’ contribute more than infectious diseases to the global burden of disease [9]. On this basis, ‘mental illnesses’ are presented as ‘killer diseases’ that ‘need to take their place among the other killer diseases for investment and priority’ [11]. As well as comparing disease burden, the economic burden of (and return on investment in) mental health and communicable diseases (including HIV/AIDS) are often compared [12]. It has been argued that treatment for mental disorders is as cost-effective as antiretroviral treatment for HIV/AIDS [13, 14], and that investment in addressing mental health generates returns commensurate with investment in diseases such as HIV/AIDS [15].

As well as comparing mental health to HIV/AIDS, GMH advocacy has explicitly modeled itself on the success of HIV/AIDS advocacy. The Overseas Development Institute notes in a 2014 report that ‘The HIV/AIDS movement has made enormous progress in capturing policy-makers’ attention, attracting funding to the issue and helping to overcome associated stigma’ [16]. Gostin states that lack of social mobilization within GMH advocacy accounts for the ‘incommensurate response to mental illness when compared with HIV/AIDS’ [17]. Burns enjoins ‘the mental health community – both professional and lay… to take a leaf out of the HIV/AIDS advocacy movement’s book and mobilise itself into a high-profile, populist force to be reckoned with. Mental illness and its service-related needs must be thrust into the public eye as a major issue which, if ignored, will threaten the well-being of society in all its facets – social, economic and political’ [18]. Kleinman calls for ‘recognition that any effective change in global mental health will have to prioritise moral transformation as the foundation for reform of global mental health, much as it was for the reform that spurred HIV/AIDS treatment in Africa and Asia’ [19].

It should be noted that although the MGMH claims the title of a singular ‘movement’ there is a trifurcation in the global mental health field. There is the MGMH, which, while largely originating from professional-led advocacy, now includes members who identify as psychosocially disabled and global NGO members. There are also those organizations that advocate for global mental health in a related but distinct sense from the ‘Movement’, such as the WHO and the World Bank. Finally, there are organized movements of activists composed especially of users/survivors of psychiatric systems, and ‘Mad Pride’ activists, who organize across national borders to resist the over-extension of psychiatric power, connecting psychiatry’s colonial history with contemporary forwarding of western psychiatric ‘solutions’ as appropriate in LMICs. Examples include Transforming Communities for Inclusion, the World Network of Users and Survivors of Psychiatry, and MindFreedom, amongst others. Although there is some overlap between these three areas of action, we refer to the MGMH (in relation to the first type of advocacy), to GMH advocacy (to refer to a wider mobilization, including the WHO) and to user/survivor activists in reference to the third kind of action. While the MGMH and other GMH advocates have proposed HIV/AIDS advocacy as a model for securing resources for mental health, user/survivor activists have not generally pursued this line of argument precisely because they often resist the furthering of Western medical models of ‘mental illness’ globally.

The remainder of this article presents a debate on four potential pitfalls of comparing HIV/AIDS and mental illness and/or adopting the model of HIV/AIDS advocacy in GMH advocacy (especially in an over-simplified form), while also pointing to some possibilities for more productive engagement between advocates, activists, and scholars in both fields.

## Discussion

Recent successes in getting mental health on the global health and international development agendas have relied on the two-pronged strategy of:

1. likening mental health to infectious disease and;
2. explicitly modeling GMH advocacy on HIV/AIDS advocacy.

Now that mental health is increasingly on the agenda (for example, in the SDGs), GMH advocates continue to use these strategies to push for attention and resources. Learning from previously successful advocacy movements might seem to be a sensible approach. However, claiming HIV/AIDS as a model for global mental health advocacy raises potential problems.

As regards the first prong, we argue that infectious disease and mental health involve differing political and ethical issues that limit how far comparisons can be made between one and the other. The problems with this comparison only grow worse when the diverse class of experiences referred to as mental illness is compared to a single infectious disease like HIV/AIDS.

As regards the second prong, we argue that the comparisons that are drawn between GMH advocacy on the one hand, and HIV/AIDS advocacy on the other, whilst superficially plausible, are often based on ‘broad brush’ simplifications or misconstructions of the history of HIV/AIDS advocacy. There is a tendency to overlook important recent developments in the HIV/AIDS field– in particular the move away from seeing HIV/AIDS as a single ‘global’ problem towards focusing instead on the local contexts in which the disease is contracted.

Given these problems, it is timely and essential to consider what, if any, lessons should be learned by GMH advocates from HIV/AIDS. In particular, we argue that paying more attention to the struggles of HIV/AIDS advocacy as well as to its successes may be particularly useful.

In considering this comparison, we build upon work that has examined the different ways in which health issues have been ‘framed’ to capture political attention and resources. ‘Framing’ is of vital importance to the construction of global priorities, and for how global public health is defined and delivered [20–22]. Scholars have noted that global health issues have been variously framed in terms of human rights [23], international security [24–30], international development [31], or in terms of evidence-based medicine [32]. Here we seek to extend this analysis to examine not only how health is framed in relation to external goods (human rights, security, etc), but also to examine how health issues have been framed in relation to each other: in this case, how mental health is framed in relation to HIV/AIDS.

We outline four key problems with the model of GMH advocacy that is being developed through these analogies: 1. Issues with a universalizing global approach to context-specific problems; 2. Issues with a conception of human rights that focuses on the right to access treatment at the expense of the right to refuse it; 3. Issues with treating poverty as a psychiatric issue, rather than recognizing that mental distress can be the result of poverty and other forms of inequality; 4. The prioritization of destigmatization of disease over anti-discrimination or social justice models.

### Mental health as a ‘global’ problem: learning from HIV/AIDS advocacy

AIDS has been credited with having ‘invented’ global health, building ‘the foundation for a revolution that upended traditional approaches to “international health,” replacing them with innovative global approaches to disease’ [33]. Certainly the rise of global health as a policy field has been closely related to the rise in political commitment to tackling HIV/AIDS. Crucial to the generation of that political commitment was the argument that HIV/AIDS was a genuinely *global* challenge, affecting rich and poor societies alike, for which the solutions needed to be similarly global. In the same way, those advocating for GMH conceptualise mental disorders as ‘truly universal…found in people of all regions…at all stages of the life course’ [8], indeed that ‘mental health problems are extremely common in all countries of the world’ [34].

This framing of ‘mental health’ as a global problem is premised on the notion that there is universal consensus on what constitutes a ‘mental disorder’, and on diagnostic criteria; that mental distress is an ‘illness’; that the prevalence of mental disorders can be objectively measured in a similar way to infectious disease; and that the multiplicitous field covered by ‘mental illness’ is susceptible to being tackled by a unified strategy. Yet no such global consensus exists on these premises [35–38].

Whilst there are certain diagnostic models that purport to be authoritative (most notably, the Diagnostic and Statistical Manual - DSM; and the International Classification of Mental and Behavioral Disorders - ICD) even these function as a regularly-revised summary of often sharp professional disagreements about what should be included and how it should be defined, rather than a stable consensus. Consensus groups have been formed for a number of mental disorders (namely ADHD and Depression). Yet these have been critiqued for being influenced by the pharmaceutical industry [39], and for forestalling debate about the validity of various diagnoses, cultural constructions of ‘normal’ behaviour, global applicability of diagnostic systems, and the efficacy of treatment [37]. Attempts at international consensus often overlook widespread concerns about the deleterious effects of prolonged use of psychotropic drugs - the hallmark of current bio-medical psychiatry [40–44]. Furthermore, there is also ‘currently no accepted consensus on what constitutes positive outcome for individuals with mental illness’ (where eradication of ‘symptoms’ does not always improve in parallel to social functioning) [38].

Beyond this, it is also notable that the models and organisations that claim authority are invariably based in high income countries (HICs), usually western, and that the explanatory model of mental illness they articulate is drawn from study of populations of usually high-income countries. LMICs have a variety of different ways of naming, understanding and responding to similar affective phenomena and often actively resist the implementation of western models of mental health [45]. Thus, many have questioned the assumptions embedded in models that claim to be global, in particular that they promote simplistic biomedical frameworks disconnected from lived experience, and overlook indigenous forms of healing, social support networks and rights-based organizing [36].

Many professionals make the case for a paradigm change within psychiatry based on evidence that, in summary, psychiatric diagnoses are not valid, do not aid treatment decisions, impose Western beliefs about mental distress on other cultures [35], may increase stigma [46, 47], and are sites of institutional racism in many multicultural HICs [48].

Wherever one is positioned within this debate it is indisputable that there is ongoing controversy about the nature of mental illness, and whether the experiences it describes can even be understood as “illnesses” at all. This kind of debate is not the case with HIV/AIDS, or even infectious disease more generally.

There are several further points of debate which serve as reasons to be cautious about viewing mental health in such universalizing ‘global’ terms. The ‘global’ approach can overlook regional-level priorities and varying colonial histories, and it can also obscure how global economic and political forces, such as externally imposed neo-liberal restructuring of economies and health systems, impact experiences of mental health and well-being [49].

Rather than assuming that mental health is a universal or global problem, much could be learned by the GMH community from a more careful engagement with contemporary thinking on HIV/AIDS. Firstly, critical analysis of HIV/AIDS has examined the ways that scales of local-global are used to assert power, with influential ‘global’ movements framing particular disorders as ‘global’ to shape the terms of intervention [50, 51]. Secondly, over the last 20 years HIV/AIDS policy communities have become increasingly nuanced in the ways in which they have understood HIV/AIDS as ‘global’, shifting towards a much more fine-grained understanding of the differences between the epidemics ongoing in different parts of the world. As Wilson and Halperin argue*:*

The quest to better understand epidemics reflects growing recognition that there is no single global HIV epidemic, but rather a multitude of diverse epidemics. No single prescription can apply to countries as diverse as South Africa, Egypt, Russia, Thailand, or Papua New Guinea. The era of standard global prevention guidance is over. [52]

This insight could prove doubly relevant to the field of mental health, which covers a significantly wider field of disorders than that captured by HIV/AIDS and which moreover (as already noted) lacks universal agreement on the validity of psychiatric disorders, or even whether the very notion of psychiatric disorders aptly captures experiences of mental distress or differences in cognition.

Thus far, GMH advocacy appears to have failed to take on board lessons concerning the move away from universalizing global guidance evident in the HIV/AIDS case. Instead, global guidelines, such as the WHO’s Mental Health Gap Action Programme (mhGAP) [53], and algorithmic diagnostic tools, such as the WHO’s mhGAP Intervention Guide [54], have been specifically developed to aid treatment decisions in LMICs, and according to advocates, ‘should become the standard approach for all countries and health sectors’ [55]. GMH advocates have adopted a model of HIV advocacy based on ‘globality’ that has since been questioned by HIV advocates, practitioners, and scholars in that field. A more nuanced and up-to-date approach could fruitfully guide GMH policy in a more localized direction.

### Human rights and access to treatment

Human rights have been at the forefront of HIV/AIDS advocacy. Yet as medicines for both HIV/AIDS and ‘mental illness’ have become embedded within global and public health, some have commented on the rhetorical slippage from the ‘right to health’ to a ‘right to access treatment’ which implicitly equates health with consumption of pharmaceuticals [56]. We question whether the ‘right to access treatment’ argument can be straightforwardly imported from HIV/AIDS advocacy (where it has been hugely influential) to mental health, especially on a global scale.

One key early article written by three major MGMH figures asserts that HIV advocates recognized the need to argue that:

persons with HIV/AIDS in developing countries had the right to access antiretroviral drugs, that the state had to provide them for free, that drug companies had to reduce their prices…that apparently complex treatment regimens could be provided by primary health care providers… These arguments were moral and human rights based….We believe that the time is ripe for such a global mental health advocacy initiative that makes the *moral case* for the mentally ill ([57], emphasis added).

This is rhetorically powerful but on closer inspection difficulties arise, firstly in constructing the roll-out of AIDS medicines purely as a matter of moral action, and secondly in asserting that treatment for ‘mental illness’ can be similarly cast as a moral imperative.

First, this understanding of the prioritization of AIDS as a victory for human rights advocacy glosses over the fact that HIV/AIDS’ priority status was not entirely the product of moral compulsion: US policy-makers were concerned that high HIV prevalence in sub-Saharan Africa (including in African militaries) could lead to state failure, posing a threat to global stability and American national security [58–60]. This framing of HIV as a military and security issue was an important contributor to a perceived political imperative to tackle the pandemic. In some cases this included prioritizing the health needs of military actors.

Secondly, even in the case of HIV/AIDS, although it is routine for advocates and organizations to talk in terms of ‘prevention, treatment and care’, in practice treatment (specifically the provision of antiretroviral medications) has dominated policy and funding priorities. In low-income countries in 2013, more than twice as much was spent on treatment as prevention^1^ [61, 62]. The reasons for this focus on treatment are varied, including successful advocacy for the right to access treatment, growing evidence for the effectiveness of treatment as prevention [63], and a desire to avoid addressing the issues that in many societies make prevention activities politically and culturally sensitive [64]. In part, however, the focus on treatment also reflects broader trends of medicalization and pharmaceuticalization, in which drug-based solutions are seen as the ‘go-to’ solution for pressing health challenges [65], and healthcare rights are envisioned primarily as being rights to those drugs.

Such a narrow view of human rights, borrowed from the specific context of infectious disease, poses particular problems in GMH, where there are significant ongoing controversies concerning medicalization and pharmaceuticalization [66]. It is one thing to argue we might learn something about how to advocate for GMH from the history of advocacy for HIV/AIDS; it is another to assume that solutions in one field are appropriate in another. In the case of mental health, the problem may not be lack of access to treatment but quite the reverse: the right to access information about, and to refuse, treatments - including medications that in some cases have been found to be harmful, particularly long-term [67, 44]. There is also evidence of widespread human rights abuses within psychiatric institutions worldwide (see, for example, see the work of Mental Disability Rights International).

These rights abuses are sometimes recognized and highlighted by GMH advocates, who argue that (for the most part) care is better delivered within communities not institutions. Yet many problems exist within community care which, in both the global North and South, tends to be dominated by medication [68], sometimes resulting in forced medicating and bio-incarceration [69] and deprivations of legal capacity [70].

Globally, groups of psychiatric users and survivors, and those who identify as psychosocially disabled or mad positive, mobilize for: the right to have a choice in how they are treated; the fundamental human right to live independently and be included in the community espoused in Article 19 of the United Nations Convention on the Rights of Persons with Disabilities (UNCRPD); and the need for distress to be contextualized within social and economic conditions. See for example, the work of Bapu Trust, India; the ‘Cape Town Declaration’ of the Pan African Network of People with Psychosocial Disabilities; the World Network of Users and Survivors of Psychiatry; and MindFreedom.

Despite some recognition by GMH advocates of the social and economic determinants of mental distress, the main assumption remains that mental health is a matter of ‘illness’, similar to infectious disease, and the suggested solution remains ‘global deployment of Western biomedical models of mental disorder’, with community participation and recovery conceptualised through a narrow individualized medical framework [71]. This is insufficient.

While the ‘right to treatment’ model has been important in global efforts to tackle HIV/AIDS, it is misleading to assume it can be straightforwardly applied in the field of GMH. A more nuanced lesson that may be learned by looking at the struggles as well as the successes of HIV/AIDS advocacy is that there are pitfalls to overemphasizing treatment in framing the human right to health. Since even in the realm of infectious disease management, the emphasis on treatment has a propensity to prioritize pharmaceutical solutions over investments in robust public health solutions and health infrastructure, or tackling poverty as a social and economic (rather than a primarily medical) issue.

### Health, poverty and development

Relatedly, another debate concerning the applicability of HIV lessons to global mental health concerns the nature of the relationship between health, poverty and development. The relationship between HIV/AIDS and poverty has been portrayed as two-way; poverty exacerbates the epidemic whilst the epidemic in turn worsens inequalities [31]. HIV/AIDS advocates have made the case that expanding access to HIV services is cost effective, has macroeconomic benefits, and is a way to turn the ‘vicious cycle’ of sickness and poverty into a ‘virtuous’ one of wellbeing and economic progress [72].

The relationship between ‘mental illness’ and poverty is also often framed as a ‘vicious cycle’: ‘mental health problems are a brake on development as they cause (and are caused by) poverty’ [11, 73–75]. In practice, however, much of the focus of GMH advocates has been on how preexisting mental health problems contribute to poverty (rather than on how poverty leads to mental distress). Much GMH advocacy has argued that a high prevalence of ‘mental disorder’ constitutes a barrier to international development. Thornicroft and Patel [76], drawing upon an influential study by Bloom et al. [12], state that ‘people with untreated mental disorders have a negative effect on global wealth’ through school and work absenteeism, unemployment and healthcare costs, and impaired productivity - ‘costing the world in excess of $16tr (£9.5tr; €12tr) a year in lost economic output’. Set against this cost, mental health treatments (especially those based only on medications) look relatively cheap – estimated at between $3–9 per capita [77].

Major international programs have proceeded based on this logic of return on investment, such as the WHO MIND (Mental Health in International Development) and the UK Department for International Development (DfID) MHaPP (Mental Health and Poverty Project) [78]. In such programs, medical intervention is pitched as a solution to poverty. Some have even suggested that poverty alleviation efforts (such as microfinance) are less effective than mental health treatments in breaking this ‘cycle’ [79].

Frameworks premised on a ‘vicious cycle’ tend to prioritize economic arguments (the economic ‘burden’ of mental distress) over ones centered on justice [49] and to focus on individual change rather than systemic change as a means of tackling poverty. By positioning mental distress as a barrier to economic development, the current economic system is taken for granted and its debilitating effects on mental health recast as ‘problems’ of individual brains. This enables psychiatric and psychological expertise to be mobilized in relation to people living in poverty, contributing to the psychologization and psychiatrization of poverty.

Furthermore, attempts to measure the mental health effects of poverty are problematic if they use tools based on individual level global North diagnostic criteria, which have been critiqued for reifying psychiatric diagnostic categories that individualise distress and reconfigure ‘symptoms’ of oppression and inequality into ‘symptoms’ of mental disorder [80].

These concerns are doubly worthy of attention when we acknowledge three profound ironies. First, that just as largely medicalized mental health services and algorithmic approaches to diagnoses (see [54]) are being scaled up in the Global South, these same services are coming under enormous criticism in the Global North [35]. Second, that the WHO found better outcomes (as defined by the WHO) for people diagnosed with Schizophrenia in ‘developing’ countries, such as India and Nigeria, than in high-income countries [81, 82]. Third, that the deployment of economic-based arguments have come in for heavy criticism from critical global health scholars [83, 84] – not least as they have been applied to HIV/AIDS [31]. As such, arguments that draw on the history of HIV/AIDS to place individuals’ mental states and psychology – rather than political or economic systems – at the center of proposed solutions to global inequality and poverty are worthy of skepticism.

### De-Stigmatization, anti-discrimination, and social justice

GMH advocates argue that just as HIV advocates fought for the de-stigmatization of people living with HIV, so too should GMH initiatives foreground the de-stigmatization of ‘mental illness’. However, the application of the infectious disease model of de-stigmatization is debatable in the field of GMH, especially when it proceeds without also incorporating anti-discrimination and social justice work.

Combatting both stigma and discrimination has been at the heart of HIV/AIDS advocacy since the 1980s, in no small part as a result of pre-existing discrimination against those who were (and continue to be) at higher risk, including men who have sex with men, intravenous drug users and sex workers. Advocates have often highlighted the ways in which stigma can discourage people from seeking testing and treatment; can lead to social and economic isolation; and is connected with broader systems of discrimination, such as homophobia and criminalization.

Within GMH, stigma is understood as ‘problems of knowledge (ignorance), attitudes (prejudice) and behaviour (discrimination)’ [85] that act as barriers to treatment and to mental health gaining policy traction [16]. GMH advocates have noted that ‘knowledge about HIV/AIDS was the most powerful tool to combat stigma’ [57]. By this logic, knowledge about mental health is imagined to combat stigma, where knowledge is understood through a disease model.

This framing shores up the authority of medical experts to make decisions about people’s lives, and contradicts user/survivor activism that seeks to destigmatize madness as a form of difference, or to highlight the social and economic contexts in which distress is produced.

Moreover, research has found that disease-based and biological explanations for mental distress are *more* stigmatizing and *more* likely to increase public desire for distance than psychosocial explanations (such as distress as response to trauma or difficult living conditions) [47]*.*

Further, the disease analogy tends to emphasize the de-stigmatization of disease (i.e. the idea that the ‘disease’ of ‘mental illness’ should not be stigmatized) above all else. Anti-discrimination is pursued solely through attempts to normalize mental disorder (often through emphasizing high prevalence) and to reduce, for example, employment-based discrimination of those with a mental health diagnosis.

While this focus is valuable, it is also insufficient. GMH advocacy could learn from HIV activism concerning de-stigmatization, anti-discrimination, and social justice. HIV activists have historically taken a more holistic approach, focused not only on discrimination on the grounds of serostatus, but also the interconnected nature of different forms of discrimination, including racism, homophobia and criminalization. This sort of rounded approach to social justice is sorely needed in mental health, given the well-documented racialization and gendered nature of many mental health diagnoses, and evidence of the institutional racism of psychiatry [48].

Imagine if GMH based its de-stigmatization work in robust and holistic social justice frameworks, opposing psychiatric incarceration, emphasizing self-determination, and committing itself to tackling global inequality as a matter of justice, rather than problematizing individual behaviours of people living in poverty. This might enable much wider structural forms of advocacy around mental health, for example, by recognizing not only work-based discrimination against mental disorder but also how free-market demands of limitless growth, increased productivity and resultant unsafe and insecure working conditions may lead to mental distress, and so dedicating resources to opposing insecure working conditions as a kind of preventative action. Enhancing the representation of people experiencing mental distress would also be an important part of such a social justice-based framework.

In answering the question ‘what can advocates of mental health service transformation learn from the extraordinary success of the HIV/AIDS advocacy movement?’, Burns states that the first lesson is that successful advocacy ‘must, at its core, be represented and driven by ‘user activists’ – that is, individuals living with HIV, mental illness, etc’ [18]. In the HIV/AIDS field, the principle of the Greater Involvement of People Living with HIV/AIDS (GIPA) has been a much-vaunted accomplishment [86, 87]. Whilst imperfectly applied in many instances, the principle that People Living with HIV/AIDS should be involved in decision-making *at all levels* – from the local health facility up to global institutions – has done much to enhance both the quality and the legitimacy of decision-making. It stands as an example to GMH advocates of a practical way in which steps towards greater justice can be pursued within formal institutional arrangements.

## Conclusions

What are the effects of adopting HIV/AIDS advocacy as a model for GMH policy and action? What kinds of understanding and action does the analogy enable? Where do we draw the line between making appropriate analogies between GMH advocacy and HIV/AIDS advocacy, and making inappropriate analogies between mental health and HIV/AIDS? What version of HIV/AIDS advocacy is being adopted, and which facets of HIV/AIDS activism are being ignored, simplified or miscast?

While much scholarly literature has assessed the merits and potential problems with framing health issues in relation to social goods such as human rights, security, or development, this article has debated the advantages and disadvantages of framing health issues in relation to each other, and specifically of importing models from HIV/AIDS to make claims about mental health. Whilst we have highlighted four crucial problems with this framing, we have also argued HIV/AIDS advocacy can provide GMH with both useful lessons and also some cautionary tales. There is much to learn from a more sustained and careful engagement between HIV and GMH advocacy. Such an engagement would pay attention to the struggles of HIV/AIDS advocacy as well as its successes, take into account issues of local/global dynamics, human rights, poverty, anti-discrimination, and justice, to name a few, and listen to the voices of those with lived experience. Among the lessons it might provide, we can suggest three:

First, we need to conceptualize disease and distress as multiple and localized epidemics, responding differently to distress linked to conflict, displacement, structural adjustment policies, amongst others. This is the current conclusion of work within HIV/AIDS, and it seems even more relevant to mental health, where there is a wide field of kinds and sources of distress, no (known) universal pathogen, no consensus on whether that distress is an illness, and a plethora of different explanatory models and methods of support globally. Second, we need to move away from top-down global prescriptions and emphasise not only pharmaceutical solutions but also the promotion of social justice and material well being. Third, we need to promote meaningful non-tokenistic involvement, leadership and decision-making of those who have expertise through experience (such as users and survivors).

With these lessons in mind, there is indeed great potential for learning and cross-pollination of strategies across communities struggling for and within systems of global public health.

## Abbreviations

GIPA: Greater involvement of people living with HIV/AIDS
GMH: Global Mental Health
HIC: High-income country
HIV/AIDS: Human immunodeficiency virus/acquired immunodeficiency syndrome
LMIC: Low and middle income country
MGMH: Movement for Global Mental Health’
NGO: Non-Governmental Organization
SDGs: Sustainable development goals
WHO: World Health Organization

## Footnotes

1 45% of HIV programme spending went on treatment, as compared to 22% on prevention (17% went on ‘programme coordination’ and 16% on ‘other’)
